# Supplementary material for: Whole-cortex in situ sequencing reveals input-dependent area identity
Source: Nature. 2024 Apr 24;647(8088):203–12. doi: 10.1038/s41586-024-07221-6 (PMC12589132; doi:10.1038/s41586-024-07221-6)
Supplement: Supplementary file 1 — This file contains Supplementary Methods, Supplementary Notes 1–8 and Supplementary Table 2. [file 41586_2024_7221_MOESM1_ESM.pdf]

nature portfolio

https://doi.org/10.1038/s41586-024-07221-6

Supplementary information

Whole-cortex in situ sequencing reveals input-dependent area identity

In the format provided by the authors and unedited

Methods

Animals and tissue processing

All animal and surgical procedures were carried out in accordance with the Institutional Animal Care and Use Committee at Cold Spring Harbor Laboratory and Johns Hopkins University. The animals were housed at maximum of five in a cage on a 12-h on/12-h off light cycle. The temperature in the facility was kept at 22 °C with a range not exceeding 20.5 °C to 26 °C. Humidity was maintained at around 45–55%, not exceeding a range of 30–70%.

The animal used to generate the pilot dataset was a male 7–8-week-old C57BL/6J animal purchased from Jackson Laboratory. The four littermate pairs consisted of two male pairs and two female pairs of C57BL/6J (JAX strain no. 000664). Littermates were randomly assigned to control or the enucleation group. Sample sizes were not determined prior to the experiments. Blinding was not possible because of apparent differences in appearances between animals of the two conditions.

Bilateral enucleation surgeries were performed on mouse pups of both sexes on postnatal day (P) 1 using previously published methods [43,53,54]. Pups were anesthetized briefly with 1-2% inhaled isoflurane (Fluriso, VetOne, Boise, ID). The eyelids were opened with a sterile surgical scalpel blade. Using fine forceps, eyeballs were lifted away from the orbit and freed from the optic nerve and surrounding musculature. Following eye removal, eyelids were closed and sealed with surgical glue (Vetbond, 3M, Maplewood, MN). After surgery, pups were placed in a plastic box in a warm water bath maintained at 37°C for ~1 h for recovery before returning to their mothers. Only one-half of the entire litter underwent enucleation surgery to ensure maternal care and the thriving of the pups. Sham surgery was performed as the control on the other half of the litter at the same age, where pups were subjected to anesthesia and revival procedures as described above. All pups were housed with their mothers until they were used for experiments at P28 and were weighed routinely to ensure normal thriving.

To collect brains for BARseq, we euthanized the animals with isoflurane overdose, decapitated the animals, embedded the brain in OCT and snap-froze in an ethanol dry-ice bath. In experiments in which only one hemisphere was used, we bisected the brain along the midline before OCT embedding. The brains were then sliced to 20 µm hemi-coronal sections and mounted onto Superfrost Plus Gold slides. Eight sections were mounted onto each slide.

BARseq library preparation

BARseq was performed on 40 hemi-coronal sections in the pilot dataset, and 32 hemi-coronal sections per animal in the enucleation experiment. The 32 sections were collected starting from the posterior end of the cortex with 200 µm spacing between each collected section, which ensured that they captured the whole visual area in all animals.

BARseq samples were prepared as previously described [14]. Briefly, slides were fixed in PFA, dehydrated in ethanol, rehydrated in PBST (PBS with 0.5% Tween-20), then reverse transcribed using random primers with a N-terminal amine group and Revert-aid H-minus reverse transcriptase (Thermo Fisher). On the second day, we crosslinked the cDNA products, then performed padlock probe hybridization and ligation, followed by rolling circle amplification with ϕ29 DNA polymerase (Thermo Fisher). On the third day, we crosslinked rolonies. To sequence the samples, we hybridized sequencing primers and manually performed sequencing using either the Illumina HiSeq Rapid SBS kit v2 (for the pilot dataset) or the Illumina Miseq nano v2 kit (for the eight littermates) following previous protocols [14]. After seven sequencing cycles were

completed, we then striped the sequencing primers using three incubations at 60°C for 5 mins each in 60% formamide, 2× SSC, 0.1% Tween 20. We then hybridized fluorescent probes in 10% formamide, 2× SSC for 10 mins, followed by incubation in 2 μg/mL DAPI in PBST for 5 mins. We then imaged the final hybridization cycle. Detailed protocols are available at protocols.io: dx.doi.org/10.17504/protocols.io.81wgbp4j3vpk/v2. Primers and padlock probes used are listed in **Supplementary Table 1**.

BARseq data collection

BARseq data from the pilot brain were collected as described previously [14] on a Olympus IX-81 microscope with PI P-736 piezo z-stage, Olympus UCPLFLN20X 20× 0.7NA air objective, a Crest xlight v2 spinning disk confocal, 89North LDI-7 laser bank, and Photometrics BSI-prime camera. The microscope was controlled using micromanager v1.4. Because the microscope stage could only fit 3 slides at a time, the posterior 22 sections and the anterior 18 sections were collected in two separate batches. BARseq data from the eight littermate brains were collected on a Nikon Ti-2E microscope with PI P-736 piezo z-stage, Nikon CFI S Plan Fluor LWD 20XC 0.7NA objective, a Crest xlight v3 spinning disk confocal, Lumencor Celesta 7-line laser, and a Photometrics Kinetix camera. The microscope was controlled using NIS-Elements 5.30.04. Four slides with eight slices per slide, all from the same animal, were imaged in each batch. The filters and lasers used are listed in **Supplementary Table 2**.

BARseq data processing

BARseq data were processed as described previously[14] with slight modifications. Briefly, we created max-projections from the image stacks, applied noise reduction using Noise2Void [55], applied background subtraction, corrected for channel shift and bleed-through, and registered images through all sequencing cycles. We segmented cells using Cellpose [56], decoded rolonies using BarDensr [45], and assigned rolonies to cells. During BarDensr decoding, we used negative control GIIs (i.e., GIIs that were not carried by any padlock probe) to estimate false discovery rate (FDR), and automatically adjusted decoding threshold to target about 5% FDR. Finally, the images were stitched to generate whole-slice images. All processing steps were performed on each imaging field of view (FOV) separately, not on the stitched images, to avoid stitching errors, to minimize alignment artifacts due to imperfect optics, and to facilitate parallel processing. The stitched images were only used to generate a transformation, which we applied to each rolony and cell after all other steps were finished. Overlapping cells from neighboring FOVs were detected using a custom implementation of sort and sweep [57], an algorithm that is commonly used in detecting object collision in video games. When two or more cells were detected as overlapping, cells that had more read counts (which we assumed to have higher read quality) were kept, and all other cells were removed. See **Data availability** and **Code availability** for processing script and intermediate processed data.

We then registered each stitched slice to Allen CCF. For each slice, we first generated an image that color coded each cell by its H2 type identity. We then used QuickNii (v.3 2017) to manually select the CCF plane for each slice and Visualign (v. 0.9) to align area borders within each slice. After we obtained CCF coordinates for each neuron, we registered all cortical cells onto a CCF flat map using the python ccf_streamlines package (https://pypi.org/project/ccf-streamlines/). Streamlines represent the paths that most directly connect the pia of the isocortex to the white matter while following the curvature of those surfaces. Because the streamlines were defined in CCF space and are thus consistent across all animals, this

approach allowed us to obtain relative cortical depth and relative cortical location of each neuron more reliably across the eight brains compared to the manual approach used in the pilot brain.

Quality control

After segmentation, we obtained a total of 13,886,988 segmented cells. We kept all cells expressing at least 5 unique genes and at least 20 total counts, resulting in a count matrix with 104 genes and 10,378,092 cells.

Iterative clustering and annotation of BARseq transcriptomic data

Generally, two approaches can be used to identify cell types in new transcriptomic datasets. In the first approach, we can map individual neurons in a new dataset directly to clusters in reference single-cell RNAseq datasets to determine cell type identities. This approach can match small datasets to cell types discovered in much larger, higher-resolution datasets [58-60], but is prone to technique-specific variations when mapping data generated by different techniques [61]. Alternatively, we can cluster the new dataset and map *clusters* to cell types in reference single-cell RNAseq datasets [8,36]. This approach can better account for technique-specific variations and batch effects [44], but the ability to distinguish cell types is dependent on both data quality and sample size in the new dataset. Because our pilot dataset contained 1.2 million cells, which is comparable in size to many comprehensive single-cell RNAseq datasets [9,26,32], we reasoned that *de novo* clustering followed by assessment at the cluster level would be more easily interpretable.

To obtain H1, H2, and H3 types in the pilot brain, we adopted an iterative clustering pipeline adapted from single-cell RNA sequencing (scRNAseq) studies [62]. We performed 3 rounds of clustering with the following steps: normalization, dimension reduction (PCA), computation of a shared nearest neighbor (SNN) network, and Louvain clustering. We normalized counts to CP10 (counts per 10) values, then applied the log1p transformation (log with a pseudo-count of 1). Because our panel consists of marker genes, we skipped highly variable gene selection and ran PCA on all genes. We computed PCA using the scater::runPCA function [63] and kept the first 30 PCs. We built the SNN network using the scran::buildSNNGraph function with 15 nearest neighbors and the "rank" metric. We ran the Louvain clustering as implemented in the igraph::cluster_louvain function with default parameters. All UMAP visualizations were obtained using the scater::runUMAP function starting from the PCA dimension reduction and using the 15 nearest neighbors.

In the first round of clustering, we separated cells in three classes reflecting neurotransmitter expression: excitatory (expressing *Slc17a7*), inhibitory (expressing *Gad1*), and others (expressing neither *Slc17a7* nor *Gad1*). Because marker genes are frequently undetected at the single-cell level, we ran the clustering pipeline on all cells, obtaining 24 clusters, then assessed marker expression at the cluster level. From the UMAP visualization, we distinguished 3 groups of clusters. The first group contained excitatory clusters expressing *Slc17a7*, the second group contained inhibitory clusters expressing *Gad1*, the third group expressed neither of these markers. Based on these observations, we manually annotated the clusters as "excitatory", "inhibitory", and "other", respectively (H1 types, 642,340, 427,939, and 188,977 cells, respectively). Previous studies estimated the fraction of inhibitory neurons in the mouse cortex to be between 10% and 20% [64,65]. Consistent with these estimates, 16% of neurons in the cortex were inhibitory neurons in our dataset (427,766 excitatory neurons, 83,394 inhibitory neurons, and 6,268 other cells). Because the excitatory and inhibitory neurons were defined by clustering on the expression of all genes, a small fraction of them did not have detectable *Slc17a7* (3,800 of 427,766 excitatory neurons, 0.9%) or *Gad1* expression (100 of 83,394 inhibitory neurons, 0.1%). Because we only sampled *Slc17a7* and *Gad1* for

excitatory and inhibitory neuron markers, the excitatory neurons identified were dominated by cortical neurons, although we also saw neurons in the pons and the epithalamus in this group. The third group of cells, which expressed neither *Slc17a7* nor *Gad1*, included subpopulations of subcortical neurons (e.g., the midbrain and the thalamus) and non-neuronal cells (e.g., glial cells, immune cells, and epithelial cells). We expect this group of cells to be under-sampled, because these cells may not express cortical cell type marker genes that we probed at sufficient levels to pass quality control (**ED Fig. 1G, H**).

In the second round of clustering, we extracted all cells labeled as "excitatory" and "inhibitory", then ran our pipeline again on each H1 type separately. By re-running the pipeline, the dimension reduction and clustering are better targeted at finding variability specific to either excitatory or inhibitory cells. We obtained 18 excitatory clusters and 11 inhibitory clusters, which formed the basis for our H2 types. In the third round of clustering, we re-ran the pipeline on each excitatory H2 type, obtaining roughly 5 to 6 clusters by type (117 total H3 types).

To annotate H2 and H3 types in the pilot brain, we examined the brain-wide distribution and the marker expression of H2 and H3 types. Almost all H3 types showed brain-area specificity, suggesting that the data were clustered at a biologically meaningful granularity. We annotated isocortical H2 types based on aggregate marker expression (see **Supplementary Note 1** for marker selection). We annotated non-isocortical H2 types based on the localization of cells (e.g., hippocampal areas, thalamus, entorhinal cortex). When H2 types contained a mix of isocortical and other cells (e.g., "L6 IT-like"), we split the H2 type into multiple H2 types, one containing the isocortical cells (e.g., "L6 IT"), the others containing the other cells (e.g., "PIR L6 IT-like", "AON DL"). In the end, we obtained a list of 11 cortical H2 types and 23 non-cortical H2 types. After mapping to scRNAseq reference types, we noticed that four H3 types ("PT AUD", "PT P RSP/IT-like?", "RSP DL", "CT CTX A/L-V") were assigned to incorrect H2 types ("L5 IT", "RSP DL", "L5 IT", "L6b"); we manually corrected their H2 annotation (to "PT", "PT", "RSP DL", "CT").

The eight littermate brains were processed as a single batch using the same overall procedure as the pilot brain, with the following adjustments at the H1 and H2 levels to minimize computational load. We used scater's calculatePCA and calculateUMAP functions (with external_neighbors=TRUE and BNPARAM=AnnoyParam()). We computed clusters using Seurat's FindNeighbors function[66] followed by Leiden clustering[67] using igraph::cluster_leiden (with objective_function="modularity", resolution=0.2 at the H1 level and 0.8 at the H2 level). For cell type annotation, we used a 4ombinationn of the strategy described above and top hits to pilot brain annotations using MetaNeighbor.

Mapping of BARseq types to scRNAseq reference types

To map BARseq types to reference types, we used a k-nearest neighbor (kNN) approach to label each cell according to its closest neighbors in a whole-cortex and hippocampus reference compendium [9]. First, we evaluated the accuracy of the kNN approach on a subset of the reference compendium using leave-one-cell-out cross-validation (10X MOp dataset, "Glutamatergic" cells, cortical cells labeled as "CTX" or "Car3", clusters with ≥ 50 cells, CP10K and log1p normalization). To transfer labels, we picked each cell's closest 15 neighbors using the BiocNeighbors::queryKNN function, then predicted the cell's type by taking a majority vote across the neighbors. We compared accuracies across 4 gene panels: highly variable genes (HVGs, 2000 genes selected using scran::modelGeneVar and scran::getTopHVGs), HVG selection followed by PCA (30 components, scater::runPCA), the BARseq panel (104 genes, after excluding *Gad1* and *Slc17a7*), and the BARseq panel with reads down-sampled to match BARseq sensitivity (104 genes, binomial sampling of reads, re-normalization through sample-wise ranking and scaling). For the latter gene set, reads were downsampled for each gene according to the sensitivity ratio (BARseq average counts

divided by reference average counts). For genes that had a sensitivity ratio r > 1, we oversampled reads to match BARseq sensitivity (reads multiplied by $\lfloor r \rfloor$ + binomial sampling with probability $\lfloor r \rfloor - r$).

Having validated that the kNN mapping procedure was able to assign cell types with high accuracy, we applied the same procedure (sensitivity adjustment of reference datasets, sample-wise ranking and scaling of reference and target datasets, BiocNeighbors::queryKNN with 15 neighbors) to assign a reference label to each BARseq cell. In contrast with the previous evaluation, we used all excitatory cells from the reference compendium (40 individual datasets, all "Glutamatergic" cells) and adjusted reference reads using a simplified downsampling procedure (reads multiplied by the sensitivity ratio for each gene). To compute the overlap between BARseq and reference cell types, we used the Jaccard coefficient (number of cells labeled as BARseq type b and predicted to be reference type r divided by the number of cells of type b or predicted to be type r).

We mapped both H3 types in the cortex (**Fig. 2E**) and those in the hippocampal formation (**ED Fig. 2F**) to single-cell RNAseq data. However, we do not expect perfect matching for clusters outside of the cortex, because our dataset sampled additional brain regions that were not sampled in the single-cell RNAseq data. In addition, our gene panel was optimized for cortical excitatory neurons and could miss highly differentially expressed genes in other brain regions.

We mapped H3 types from the eight littermate brains against the pilot brain and scRNAseq data from Cheng, et al. [42] using the procedure outlined above.

Variance of expression explained by H2 types, H3 types, and space

To evaluate how well H2 types, H3 types, and spatial information recapitulate the variability of expression, we performed one-way ANOVA on pseudo-bulk data. This analysis was run on a subset of data containing the 8 isocortical H2 types with isocortex-wide localization ("L2/3 IT", "L4/5 IT", "L5 IT", "L6 IT", "PT", "NP", "CT", "L6b").

We started by computing the pseudo-bulk expression matrix B_{gts}, providing expression of gene g for H3 type t in spatial bin s. We defined 540 spatial bins containing an average of 14 cells per H3 type as follows: 27 slices along the A-P axis (corresponding to slices 7 to 33), 20 bins along the un-warped M-L axis for each slice (chosen to balance the number of cells in each bin, computed independently for each slice). Slices at the anterior and posterior end of the brain were excluded because coronal sections were not perpendicular to the cortical surface at these extreme positions and would thus bias gene expression. Starting from the gene by cell count matrix C_{gc}, we have $B_{gts} = mean_{c\in t, c\in s}\ (C_{gc})$.

Next, we computed the variance explained by the 8 H2 types, 51 H3 types and 540 spatial bins by applying the one-way ANOVA formula for each gene and factor independently. Let $M = mean_{t\in H3, s\in bin}\ (B_{gts})$ be the overall average expression and $T = \sum_{t\in H3, s\in bin}(B_{gts} - M)^2$ be the total variance. For gene g, we computed the variability explained as follows:

$$VE_{space} = \sum_{s\in bin} \quad (mean_{t\in H3}\ (B_{gts}) - M)^2\ /T$$

$$VE_{H3} = \sum_{t\in H3} \quad (mean_{s\in bin}\ (B_{gts}) - M)^2\ /T$$

$$VE_{H2} = \sum_{h \in H2} (mean_{s \in bin, t \in h}(B_{gts}) - M)^2 / T$$

Because H3 types are nested factors of H2 types, the variability explained by H3 types is necessarily higher; the additional variability explained by H3 types is given by $\Delta VE = VE_{H3} - VE_{H2}$.

Extraction of recurrent spatial patterns using non-negative matrix factorization

The ANOVA analysis revealed the presence of recurrent spatial patterning across genes and H2 types. We used non-negative matrix factorization to extract these patterns. First, we defined a pseudo-bulk matrix using the same procedure as the ANOVA analysis (see above), except that we computed the pseudo-bulk matrix at the level of H2 types. Starting from the count matrix C_{gc}, we have $B_{gts} = mean_{c \in t, c \in s}(C_{gc})$, where t is one of the 8 H2 types. Here, we consider the spatial bins as features (rows), genes and types as variables (columns), resulting in a matrix with 540 rows and 848 columns. Because spatial patterns had different scales (average level of expression) across genes and H2 types, we rescaled each column using L2-normalization (squared columns sum to 1). This rescaling ensures that factors reflect recurrent patterns (seen in multiple genes and H2 types) rather than a single instance (highly expressing gene in one H2 type). This procedure (pseudo-bulking at H2 level and rescaling) can also be seen as a correction for H2 type composition (overall expression patterns are dominated by the most common or the highest expressing H2 type). We extracted 10 NMF factors using the NMF::nmf function using default parameters (Brunet algorithm), obtaining a nonnegative basis matrix W (540 bins by 10 factors) containing spatial patterns and a nonnegative coefficient matrix H (10 factors by 848 co)umns) such that B≈W.H. For later analysis, we removed 3 NMF factors that reflected obvious slice-specific batch effects (see **Supplementary Note 4**).

To identify genes associated with each NMF factor, we computed the average fraction of expression variance explained by each NMF factor. Given an NMF factor f, we computed the predicted gene expression for gene g in type t as $\widehat{B_{gt.}} = W_{.f}.H_{fgs}$, where $W_{.f}$ is the column in W representing factor f, and H_{fgs} is the coefficient associated with factor f, gene g and type t. The variance explained is then computed as $VE_t = (T - \sum_{s \in bin}(B_{gts} - \widehat{B_{gts}})^2)/T$, where $T = \sum_{s \in bin} {B_{gts}}^2$ is the total uncentered variance. We then took the average variance explained across the 8 H2 types. To estimate the null fraction of variance explained, we permuted coefficients associated with each factor (permutation of rows in H), then recomputed the average variance explained across all genes and factors. We labeled gene-NMF associations as "significant" if the average variance explained exceeded the 99th percentile of the null distribution.

To identify H3 types associated with each NMF factor, we computed two measures of association: enrichment of NMF-associated genes and correlation of spatial distribution with NMF patterns. Using the MetaMarkers package, we computed differentially expression (DE) statistics for each H3 type (1-vs-all differential expression against other H3 types from the same H2 type). We then asked whether top DE genes were enriched for NMF-associated genes (genes with significant association with a single NMF). We report the enrichment as an AUROC, asking how well expression fold changes predict NMF-associated genes. Independently, we computed the overlap between NMF patterns (columns in W) with the spatial distribution of H3 types (count of cells in bin s divided by total count) using the Spearman correlation. Because the dynamic range of the correlation coefficient depends on the sparsity of the pattern (sparser patterns tend to have a smaller range of correlation values), we report the association as the Z-scored Spearman correlation (Z-scoring across H3 types within a given H2 type and factor).

Predicting cortical areas and locations using gene expression and H3 type composition

For the pilot dataset, we first binned cells in the cortex into cubelets, which were drawn separately on each coronal section, spanned all cortical layers, and were about 100 μm – 200 μm on the M-L axis along the curvature of the cortex. To draw the cubelets so that their medial and lateral borders were perpendicular to the layers, we manually drew matching points along the top and bottom surface of the cortex, especially at locations where the curvature of the cortex is extreme (e.g., the medial part of the cortex). This step thus separates both the bottom and top surfaces of the cortex into several segments. Within each segment, we then cut the two surfaces into the same number of smaller segments of roughly equal distance. Each cubelet was then defined by connecting the ends of the small segments. Slices at the anterior and posterior end of the brain were excluded because coronal sections were not perpendicular to the cortical surface at these extreme positions and would thus bias composition of neuronal populations. Unlike the spatial bins used in the NMF analysis, which had equal cell numbers within each slice but unequal widths, the cubelets had similar widths across all slices, but not necessarily similar cell numbers (**ED Fig. 4A**).

Because the cubelets are generated within each of the large segments, they may be slightly different in size across different segments. We thus normalized both H3 type counts and gene read counts within each cubelet by the total number of cells and/or gene reads for downstream analyses. For the A-P locations of each cubelet, we used the CCF coordinates directly. For the M-L locations, we calculated an un-warped coordinate along the cortex within each coronal section as follows. We connected the centroids of adjacent cubelets and defined the un-warped distance between two cubelets as the sum of all connected lines across all cubelets between the two. We then defined the zero position along this un-warped M-L axis as the point that was closest to the point in CCF where the midline of the brain intersects the top surface of the brain. Thus, cubelets on the medial side have negative M-L coordinates, whereas those on the dorsolateral side have positive M-L coordinates.

To predict the coordinates of each cubelet, we trained random forest regression models, each with 50 trees and an in-bag-fraction of 0.5. We used 100-fold cross-validation to evaluate the performance of the models. To evaluate the prediction performance using the composition of H3 types within each H2 type, we trained similar regression models using only the relevant H3 types and evaluated performance using 5-fold cross-validation. To predict cortical area labels in CCF, we first assigned CCF area labels to each cubelet using its centroid location. We then trained random forest classifiers with 500 trees and an in-bag-fraction of 0.5 and evaluated the performance with 10-fold cross validation. All models were built in MATLAB using the TreeBagger function.

Correspondence between abrupt changes in the composition of H3 types and area borders defined by CCF

To find positions of abrupt changes in the composition of H3 types within each coronal section, we performed principal component analysis on the composition of H3 types and calculated the two-norm of the 1st derivative of the first five principal components along the un-warped M-L axis. We then convoluted the two-norm of the derivatives with a smoothing window of the shape [0.25, 0.5, 0.25]. We then looked for local peaks with prominence that was larger than half a standard deviation, and with the value at the peak higher than the median of the smoothed norm of the derivatives. Peaks were considered close to a CCF-defined border if it was within 150 μm from that border, calculated based on the CCF coordinates of the centroids of cubelets. All relevant analyses were performed in MATLAB.

Inferring cortical modules from H3 type composition

We only clustered areas with at least 7 cubelets. For each pair of areas, we built a support vector machine classifier with Lasso regularization to predict the area identity given H3 type composition. We calculated the area under the ROC curves following 5-fold cross validation. The AUROC values were used to build a distance matrix among cortical areas. We then performed Louvain community detection using this distance matrix, which generated six clusters and three areas that did not cluster with any other areas. We then built a dendrogram of the six clusters and three areas using the medians of all pairwise distances between areas from each pair of clusters and ward linkage. We then manually cut the dendrogram to generate the five modules. The cortical flatmap of modules was drawn based on that in Harris, et al. [3], and color-coded manually based on this analysis.

To test how the strength of cortical modularity, we calculated the modularity of clusters with the following perturbations: (1) Randomly shuffle cortical area labels across all cubelets. (2) Randomly assign each cubelet with an area label within 1-cubelet distance with same probabilities. That is, for each cubelet, there is an equal chance of assigning an area label of the cubelet itself, or the labels of the two cubelets adjacent to it. (3) Randomly assign each cubelet with an area label within 2-cubelet distance with the same probabilities. In addition, for the random shuffling control, we calculated modularity for both the clusters identified by Louvain clustering on the original data and the clusters identified by Louvain clustering on the shuffled data.

All relevant analyses were performed in MATLAB. The linear classifier was generated using the fitclinear function, and Louvain community detection was performed using a MATLAB implementation by Antoine Scherrer.

Gene expression changes in the enucleated animals

To assess the nature of gene expression shifts from control to enucleated animals, we first investigated the neighborhood of individual neurons. If types appeared or disappeared in enucleated animals, we would expect that the neighborhood of groups of neurons would be entirely composed of neurons of either control or enucleated animals. To avoid donor and litter effects, we only considered neighborhoods across litters (e.g., comparing neurons for an animal in littermate pair 1 only to animals in pairs 2-4). For each neuron, we picked the k = 100 closest neighbors (using the same procedure as kNN dataset mapping, sample-wise ranking and scaling followed by queryKNN), then computed the fraction of neurons from control animals.

We then used the same procedure to better understand the nature of the gene expression shift. Instead of looking at the "condition" label of each neighbor (control or enucleated), we collected the brain area labels of the top k = 20 neighbors. We aggregated these counts at the H2 level for each animal, then performed a two-sided hypergeometric test within each litter to identify brain region neighborhoods that were significantly depleted and enriched in enucleated animals. We combined p-values from individual litters into an overall p-value using Fisher's method, then corrected p-values using the FDR procedure.

Cell type composition changes in the enucleated animals

To assess how cell type composition is related to cortical areas in control and enucleated animals, we analyzed the differences in H3 type composition for each cubelet in all control and enucleated littermate brains. For the eight littermate brains, we used the flatmap mapping to define cubelets. For each slice, we

fitted a polynomial curve line using flatmap coordinates of the middle layer neurons (relative depth between 80 to 120 on a scale of 0 to 200) and used this fit to represent the midline of the cortex. We then projected neurons at all depths onto this midline based on their flatmap coordinates. After projecting all neurons, we segmented the fitted line into 50 roughly equal segments. In this way, we assigned neurons into cubelets with consistent number of neurons for the control and enucleated littermate brains.

For all cubelet-based analyses, we filtered out cubelets with damaged or warped tissue, as well as cubelets on the posterior edge of cortex that did not contain all cortical layers. Areas were assigned to each cubelet based the plurality area identity of neurons within that cubelet. UMAP plots were generated using the fraction of H3 types in each cubelet.

To test how distinguishable an area is between the enucleated and the control brains, for each cubelet we identified the 10 nearest neighbor cubelets from the same cortical area in the six animals that came from different litters. We randomly selected 20% of all available cubelets for each iteration and performed 50 iterations. For shuffled controls, we shuffled the conditions of the six brains, but cubelets within each brain retained the same condition label as other cubelets from the same brain. We then calculated AUROC based on the condition labels for the nearest neighbors. We performed t-test and used the Benjamini-Hochberg procedure to find the false discovery rate (FDR).

To test how well each area from the enucleated or the control brains maps onto areas in the control brains, for each cubelet we identified the 20 nearest neighbor cubelets from any cortical area in the three control animals that came from different litters. Then for each pair of cortical areas A and B, we then calculated AUROC for classifying cubelets from A or non-A areas in the test brain to B or non-B areas in the control brains. To test the shift of cell types within each H2 type, we performed the same test using only the compositions of H3 types that belonged to a H2 type.

Data availability

Raw sequencing images are available from the Brain Image Library (https://api.brainimagelibrary.org/web/view?bildid=ace-dim-pad, https://api.brainimagelibrary.org/web/view?bildid=ace-dim-own, https://api.brainimagelibrary.org/web/view?bildid=ace-dim-owl, https://api.brainimagelibrary.org/web/view?bildid=ace-dim-out, https://api.brainimagelibrary.org/web/view?bildid=ace-dim-orb, https://api.brainimagelibrary.org/web/view?bildid=ace-dim-old, https://api.brainimagelibrary.org/web/view?bildid=ace-dim-off, https://api.brainimagelibrary.org/web/view?bildid=ace-dim-odd, https://api.brainimagelibrary.org/web/view?bildid=ace-cry-zip).

Cell-level and rolony-level data are provided at Mendeley data (https://data.mendeley.com/datasets/8bhhk7c5n9/1 and https://data.mendeley.com/datasets/5xfzcb4kn8/1). Gene panel selection and cell type assessment were based on public data available at https://data.nemoarchive.org/biccn/lab/zeng/transcriptome/ and https://github.com/shekharlab/mouseVC. Allen CCF v3 with the 2017 annotation was downloaded from https://community.brain-map.org/t/api-allen-brain-connectivity/2988.

Code availability

Scripts used for both data processing and data analysis are provided at Mendeley data (https://data.mendeley.com/datasets/8bhhk7c5n9/1 and https://data.mendeley.com/datasets/5xfzcb4kn8/1) and on Github (**https://github.com/gillislab/barseq_analysis**).

Methods references

53 Mukherjee, D. *et al.* Early retinal deprivation crossmodally alters nascent subplate circuits and activity in the auditory cortex during the precritical period. *Cereb Cortex* **33**, 9038-9053 (2023). **https://doi.org:10.1093/cercor/bhad180**

54 Deng, R., Kao, J. P. Y. & Kanold, P. O. Aberrant development of excitatory circuits to inhibitory neurons in the primary visual cortex after neonatal binocular enucleation. *Sci Rep* **11**, 3163 (2021). **https://doi.org:10.1038/s41598-021-82679-2**

55 Krull, A., Buchholz, T.-O. & Jug, F. Noise2Void - Learning Denoising from Single Noisy Images. arXiv:1811.10980 (2018). <**https://ui.adsabs.harvard.edu/abs/2018arXiv181110980K**>.

56 Stringer, C., Wang, T., Michaelos, M. & Pachitariu, M. Cellpose: a generalist algorithm for cellular segmentation. *bioRxiv*, 2020.2002.2002.931238 (2020). **https://doi.org:10.1101/2020.02.02.931238**

57 Baraff, D. & Witkin, A. Dynamic simulation of non-penetrating flexible bodies. *SIGGRAPH Comput. Graph.* **26**, 303–308 (1992). **https://doi.org:10.1145/142920.134084**

58 Cadwell, C. R. *et al.* Electrophysiological, transcriptomic and morphologic profiling of single neurons using Patch-seq. *Nat Biotechnol* **34**, 199-203 (2016). **https://doi.org:10.1038/nbt.3445**

59 Scala, F. *et al.* Phenotypic variation of transcriptomic cell types in mouse motor cortex. *Nature* **598**, 144-150 (2021). **https://doi.org:10.1038/s41586-020-2907-3**

60 Bakken, T. E. *et al.* Comparative cellular analysis of motor cortex in human, marmoset and mouse. *Nature* **598**, 111-119 (2021). **https://doi.org:10.1038/s41586-021-03465-8**

61 Liu, J. *et al.* Concordance of MERFISH Spatial Transcriptomics with Bulk and Single-cell RNA Sequencing. *bioRxiv*, 2022.2003.2004.483068 (2022). **https://doi.org:10.1101/2022.03.04.483068**

62 Amezquita, R. A. *et al.* Orchestrating single-cell analysis with Bioconductor. *Nat Methods* **17**, 137-145 (2020). **https://doi.org:10.1038/s41592-019-0654-x**

63 McCarthy, D. J., Campbell, K. R., Lun, A. T. & Wills, Q. F. Scater: pre-processing, quality control, normalization and visualization of single-cell RNA-seq data in R. *Bioinformatics* **33**, 1179-1186 (2017). **https://doi.org:10.1093/bioinformatics/btw777**

64 Meyer, H. S. *et al.* Inhibitory interneurons in a cortical column form hot zones of inhibition in layers 2 and 5A. *Proc Natl Acad Sci U S A* **108**, 16807-16812 (2011). **https://doi.org:10.1073/pnas.1113648108**

65 Sahara, S., Yanagawa, Y., O'Leary, D. D. & Stevens, C. F. The fraction of cortical GABAergic neurons is constant from near the start of cortical neurogenesis to adulthood. *J Neurosci* **32**, 4755-4761 (2012). **https://doi.org:10.1523/JNEUROSCI.6412-11.2012**

66 Stuart, T. *et al.* Comprehensive Integration of Single-Cell Data. *Cell* **177**, 1888-1902 e1821 (2019). **https://doi.org:10.1016/j.cell.2019.05.031**

67 Traag, V. A., Waltman, L. & van Eck, N. J. From Louvain to Leiden: guaranteeing well-connected communities. *Sci Rep* **9**, 5233 (2019). **https://doi.org:10.1038/s41598-019-41695-z**

Supplementary Notes

Supplementary Note 1: Gene panel selection and overview of BARseq strategy

We selected marker genes based on 47 sets of single-cell RNAseq datasets collected in adult mouse brains, including seven datasets in the motor cortex (MOp) using different single-cell RNAseq techniques [8] and 40 datasets in the whole cortex and hippocampus using SmartSeq and 10x v3[9]. Multiple datasets allowed us to pick genes that were differentially expressed across cell types consistently across techniques. This consistency thus increased the likelihood that it would also be differentially expressed when detected using BARseq, which has different constraints and limitations from all single-cell RNAseq techniques. We focused on picking highly expressed combinatorial marker genes with large fold change between cell types instead of binary markers. Specifically, we automatically aggregated differential expression statistics using MetaMarkers [68], then restricted panel selection to genes with an average AUROC>0.8, an average detection rate (fraction of cells expressing the gene)>0.9, and an average fold change>2. We then manually selected potential gene panels based on observed expression patterns. We iterated through several panel designs with increasing numbers of genes and assessed their cell typing power using published single-cell RNAseq data. Specifically, we applied MetaNeighbor to a surrogate dataset that had comparable sensitivity to BARseq (10x v2 single-nuclei RNAseq from the MOp) and computed our ability to distinguish existing cell types (cell type separability, MetaNeighbor with 5-fold cross-validation, **ED Fig. 1A, C**) and our ability to retrieve cell types through de novo clustering (cell type clusterability, CPM-log1p normalization, kNN-Louvain clustering, MetaNeighbor against other MOp datasets, **ED Fig. 1B**). Increasing the number of genes generally improved cell typing performance, which largely plateaued with the 104-gene panel we used.

We further experimentally assessed whether the 104-gene panel (**Supplementary Table 1**) was sufficient. To do so, we performed BARseq to interrogate a 137-gene panel on two coronal sections; this gene panel included the whole 104-gene panel and an additional 33 genes that were selected following similar criteria. We then compared clusters obtained using either all genes, or the 104-gene subset from the same dataset. We found that the two gene panels resolved similar clusters at the H2 and H3 levels, and the separation of clusters was also similar (**ED Fig. 1D-F**). We thus concluded that the 104-gene panel was sufficiently optimized for resolving cortical excitatory types in the current experiments.

We used up to 12 padlock probes to target each gene; each probe carried a 7-nt gene identification index (GII) that uniquely identified the gene. These GIIs were designed to have a minimum hamming distance of 3-nt to allow for error correction. We further included 5 blank GIIs that were not present in the padlock probes as negative controls when decoding the GIIs; these blank GIIs allowed us to control and evaluate false detection rates. We decoded the GIIs from seven rounds of sequencing using BarDensr [45] while maintaining an optimal false detection rate (~5% estimated using the blank GIIs). We registered our data to the Allen Mouse Brain Common Coordinate Framework v3 (CCF v3) [39] using a semi-manual procedure that utilized QuickNii, Visualign [69], and custom python scripts (see **Code availability**). Three highly expressed high-level marker genes (*Slc17a7* for excitatory neurons, *Gad1* for inhibitory neurons, and *Slc30a3* for IT neurons) were detected by hybridization rather than by sequencing (**Fig. 1B**). We segmented cell bodies with Cellpose using DAPI as the nucleic channel in Cellpose and sequencing signals from all imaging channels as the cytoplasmic channel in Cellpose [56] (**Fig. 1B**).

Many canonical cell type markers showed expression patterns that were consistent with the patterns of *in situ* hybridization in the Allen Brain Atlas [30](**ED Fig. 1I**). For example, classical cortical layer-specific markers, including *Cux2*, *Fezf2*, and *Foxp2*, were expressed in layer 2/3, layer 5/layer 6, and layer 6, respectively. *Rorb*, a layer 4 marker, was seen in layer 4 throughout the cortex except in the motor cortex

and medial areas, which lack a classically defined layer 4. *Scnn1a* was expressed mostly in the retrosplenial cortex and primary sensory areas, with the strongest expression in the primary visual cortex and primary somatosensory cortex.

Supplementary Note 2: Estimation of cell segmentation errors

Segmentation errors in *in situ* sequencing can produce artifacts in single-cell gene expression in at least two ways. First, segmentation may exclude a portion of a cell. This type of error results in subsampling of all transcripts in the cell and can potentially result in lower quality single-cell data. To exclude these low-quality cells, we use a genes-per-cell threshold of 5 and a counts-per-cell threshold of 20 to remove cells with insufficient gene expression data. Second, segmentation may combine two neighboring cells into one cell, which is reminiscent of doublets in single-cell RNAseq experiments. To estimate doublet rate, we examined the expression of *Gad1* and *Slc17a7*, two highly expressed and mutually exclusive marker genes for cortical inhibitory neurons and cortical excitatory neurons, respectively, in both inhibitory neurons and cortical excitatory neurons. Consistent with the mutual exclusivity, the excitatory and inhibitory neurons showed distinct distributions of these two genes (**ED Fig. 2B, C**). We first calculated the median expression level for each marker gene in their corresponding cell class. We then asked what fraction of cells in the two H1 types had expression that was equal to or higher than this median expression level. The doublet rate is defined as the ratio between the fractions of cells with above-median expression in the two H1 types, divided by the fraction of the H1 type in which the marker is normally expressed [i.e. $P(Gad1 > median(Gad1)|Excitatory)/P(Gad1 > median(Gad1)|Inhibitory)/P(Inhibitory/All\ neurons)$ and $P(Slc17a7 > median(Slc17a7)|Inhibitory)/P(Slc17a7 > median(Slc17a7)|Excitatory)/P(Excitatory/All\ neurons)$]. The estimated doublet rate is 7% based on *Slc17a7*, and 5% based on *Gad1*. The value based on *Slc17a7* is likely an overestimate, because many *Slc17a7* transcripts are found outside of neuronal somata, which could inflate the estimated doublet rate.

In *in situ* sequencing experiments, the doublet rate is heavily dependent on cellular density and may vary widely across brain regions. Thus, our estimates of doublets, which were based on cortical neurons, may not reflect doublet rates in other brain regions. However, because our analyses focused on the cortex, our cortex-based estimates were appropriate.

Supplementary Note 3: Assessing differential expression of marker genes across H2 types and laminar distribution of H2/H3 types

We used MetaMarkers to identify the most differentially expressed markers across H2 types (**Fig. 2D**). Reassuringly, many identified marker genes for H2 types coincided with marker genes identified in previous studies. Whenever possible, we showed previously identified markers in three studies, which we abbreviated as T18[12], Y21a[8], and Y21b[9]. The other markers were identified as strong markers during panel selection (see **Supplementary Note 1**), and were labeled as "strong panel genes", along with the dataset used to identify the marker. The genes we selected for **Fig. 2D** are: *Slc30a3* (known pan-IT marker, T18), *Cux2* (known L2/3-L4/5 IT marker, Y21b), *Rasgrf2* (strong L2/3 IT panel gene, based on Y21a data), *Rorb* (known L4/5 IT marker, Y21a), *Etv1* (known pan-L5 marker - L5 IT, NP, PT, Y21b), *Scnn1a* (known deep layer RSP marker, Y21b), *C1ql3* (strong L6 IT panel gene, based on Y21a data), *Fezf2* (known non-IT marker, except L5 IT, Y21a), *Rab3c* (strong L5 ET panel gene, based on Y21a data), *Tle4* (known CT-NP-L6b marker, Y21b), *Tshz2* (known NP marker, T18, Y21b), *Foxp2* (known CT marker, T18, Y21a, Y21b),

Ctgf (known L6b marker, Y21b), *Synpr* (strong Car3 panel gene, based on Y21a data). We also noted that RSP cells have known *Tshz2* and *Slc30a3* co-expression (Y21b).

Both H2 types and H3 types were organized in an orderly fashion along the depth of the cortex. H2 types were concentrated in distinct layers, whereas H3 types within a H2 type were enriched in finer divisions within each layer ($p < 1\times10^{-61}$ using one-way ANOVA to compare the laminar positions of all H3 types within each H2 type after Bonferroni correction). For example, multiple H3 types of L2/3 IT, L4/5 IT, and L6 IT clearly occupied distinct sublayers in the somatosensory cortex (**ED Fig. 2H**). These results are consistent with previous studies using other spatial transcriptomic techniques [19,36,70] and with sublaminar differences in functional connectivity [71]. Thus, our data recapitulated the laminar organization of cortical excitatory neurons.

Supplementary Note 4: Gene expression variation across the whole cortex.

We found that variations in many genes were strongly explained by the composition of H2 types; these patterns were consistent with the cell type composition model (**Fig. 3Aa**). An example gene of this category is *Ctgf*, which is specifically expressed in L6b neurons at a consistent level across space (**ED Fig. 4D, *top***). Thus, variations in the expression (up to 80%) of *Ctgf* across space were largely explained by the fraction of L6b neurons in each bin rather than by variation in gene expression within cells. Other genes, in contrast, were largely explained by the composition of H3 types rather than the composition of H2 types (**ED Fig. 4C**, i.e. the area-specialized cell type model in **Fig. 3Ac**). Many genes in this category were also highly spatially variable (**ED Fig. 4C**, rho = 0.23), suggesting that H3 types are likely differentially distributed across the cortex (e.g. *Nnat*, marker of lateral areas, **ED Fig. 4D, *middle***). Finally, some genes displayed high spatial variability, but relatively low H2 and H3 variability. These genes were usually expressed in multiple H2 types (e.g. *Tenm3*, **ED Fig. 4D, *bottom***) and varied consistently in space across these H2 types, suggesting a general spatial gradient that is independent of H2 types. The spatial patterns of these genes were consistent with the spatial gradient model (**Fig. 3Ab**).

To extract spatial expression patterns that recurred across genes and H2 types, we applied non-negative matrix factorization (NMF) to pseudobulk expression data (one pseudobulk vector per spatial bin and H2 type), treating spatial bins as features (see **Methods**). To select a suitable number of NMF components, we progressively increased the number of components until we saw the appearance of components with negligible contributions to the variance explained (covering a small number of spatial bins, unlikely to represent robust biological signal). We identified 10 NMF components (**ED Fig. 4E**), which explained 76% of the variance in gene expression across space. Component NMF2 is found only in the anterior sections, which was sequenced in a different batch from the posterior sections. NMF2 thus reflects the batch effect between the two batches of samples. NMF6 is found mostly in posterior sections and shows clear stripe-like patterns. Because each stripe corresponds to a coronal section, and we expect gene expression to vary smoothly across adjacent coronal sections, NMF6 appears to capture differences in gene expression associated with cryo-sectioning differences across sections. Because these two components largely reflect technical variations in gene expression, we excluded them from further analyses. In addition, NMF9 is found only in the lateral edge of the most anterior sections. This area corresponds to the transition among the orbitofrontal cortex, the piriform cortex, and the endopiriform nucleus. Because of the area specificity and the continuity of its spatial distribution, this component likely reflects gene expression that is specific to this transition area. Nonetheless, because this component was found in only a small subset of spatial bins, we also excluded NMF9 from subsequent analyses.

Supplementary Note 5: cell type distribution across space

All H2 types contained at least one H3 type that was associated with NMF modules that were expressed in the medial and lateral areas (NMF 1, 3, 7, 10), and one to four H3 types that were associated with NMF modules that were expressed in subsets of the dorsal cortex, including the motor, somatosensory, and visual areas (NMF 4, 5, 8). The associations between H3 types and the NMF modules were different across H2 types, suggesting that different H2 types were specialized to different degrees at the H3 level. For example, the H3 types of L5 ET neurons had strong associations with individual NMFs, whereas H3 types of NP and L6b neurons showed little specialization within the dorsal cortex.

The H3 types also overlapped with their corresponding NMF modules in space. For example, L4/5 IT ML-P was associated with NMF5_VIS and was enriched most strongly in visual cortex; similarly, L4/5 IT P-L/LA was associated with NMF1_TEa_AUD and NMF7_SSs_AUD and was most highly enriched in auditory cortex and temporal association areas. Consistent with the expression patterns of NMF modules, many of these sets of areas are contained within cortical modules defined by inter-connectivity [3,4]. Thus, H3 types are associated with spatial gene co-expression modules and, at a coarse spatial resolution, are enriched in combinations of cortical areas that are highly interconnected.

When we examined H3 type distribution on coronal sections, we saw that the proportions of H3 types often changed abruptly near area borders defined in CCF. For example, three L4/5 IT types, including L4/5 IT UL, L4/5 IT ML-P, and L4/5 IT DL, were found in three adjacent somatosensory areas (trunk area, SSp-tr; barrel cortex, SSp-bfd; secondary somatosensory cortex, SSs; **Fig. 3F**), but the numbers of neurons of each type were distinct across the three areas. L4/5 IT UL was found in only small numbers in SSp-tr but expanded in both the number of neurons and their laminar span in the barrel cortex. Furthermore, L4/5 IT UL neurons were clustered along the mediolateral axis into structures that resembled barrels. In the secondary somatosensory cortex, the distribution of L4/5 IT UL lost the barrel-like patterns but remained present in substantial numbers. In contrast, L4/5 IT DL became more dominant relative to L4/5 IT ML-P. Similarly, in L5 ET neurons, PT CTX P was more dominant in SSp-tr, whereas PT CTX ML was found mostly in SSs (**ED Fig. 7C**). To quantify how well abrupt changes in H3 type composition corresponded to borders between all cortical areas, we identified positions where H3 type composition changed abruptly by identifying peaks in the absolute value of the first derivatives of H3 type composition along the ML axis within each slice. Consistent with the impression from images of coronal sections, 53% of peaks in the first derivatives were within 150 µm from the closest CCF border (we could not assess matching using a more stringent distance because 150 µm is already comparable to the cubelet size in this dataset). The fraction of peaks that were close to CCF borders was higher than 99% of shuffled controls (**ED Fig. 7D**).

To test how well the compositions of H3 types could predict cortical areas, we first used random forest classifiers to predict the AP and ML coordinates of each cubelet given either the total gene expression in that cubelet (**ED Fig. 7E**) or its H3 type composition (i.e. the fraction of each H3 type within a cubelet; **ED Fig. 7F**). We found that cubelet gene expression was highly predictive of locations in the cortex, capturing 94% of variance on both the AP and ML axes. The distance between the predicted and true location of a cubelet across the whole cortex was 235 ± 270 µm (median ± std) along the AP axis (spanning 5,900 µm) and 245 ± 364 µm along the ML axis (spanning 8,400 µm) (**ED Fig. 7E, G**). These prediction errors were close to the sampling frequency imposed by cubelet size (200 µm between adjacent slices on the AP axis, and 100 µm to 200 µm cubelet width on the ML axis). Strikingly, the H3 type compositions performed similarly well, capturing 89% variance on the AP axis and 92% variance on the ML axis (the prediction

errors were 312 ± 360 μm on the AP axis and 269 ± 402 μm on the ML axis, median ± std; **ED Fig. 7F, G**). Consistent with the high precision in predicting the absolute locations in the cortex, both gene expression and the composition of H3 types were highly predictive of the area labels in CCF (75% correct using gene expression and 69% correct using H3 type composition, compared to 8% in shuffled control; **Fig. 3G, H**). H3 types within a single parent H2 type were also somewhat predictive of cubelet locations, but those of H2 types in superficial layers (e.g. L2/3 IT and L4/5 IT) were generally more predictive of cubelet locations along the ML axis than those of H2 types in the deep layers (e.g. L6 IT and L6 CT) (**ED Fig. 7H, I**). The predicted maps correctly captured the locations of most cortical areas, and most of the incorrect predictions occurred along the borders of areas (**Fig. 3G**)

Supplementary Note 6: Data quality comparison across brains

To assess the consistency between the pilot brain and the four littermate pairs, we co-embedded the two datasets in the same UMAP space (**ED Fig. 8B**). The two datasets largely overlapped, but data for the four littermates were systematically "shifted" so that different clusters were more distinguishable compared to the pilot brain. This pattern of the shift between the two datasets is consistent with an overall change in sensitivity and data quality. To formally assess clusters between the two datasets, we mapped H3 cortical types between the pilot brain and the combined new dataset of eight brains (**ED Fig. 8C**) and found 1:1 or 1:2 cell type mapping between them for all clusters. Additionally, the H3 types from the eight brains were evenly distributed across all brains, indicating that H3 types were highly consistent across the eight animals (**ED Fig. 8D-F**). Thus, these results indicate that cell typing was consistent between the pilot brain and the eight brains, but that the eight brains had better data quality.

The better data quality achieved on the eight brains was not surprising and could reflect either biological differences or improvement in instrumentation. The data from the pilot brain were collected at Cold Spring Harbor Laboratory (Zador lab), and the data from the four littermate pairs were collected on an improved system at the Allen Institute (Chen lab). The improved system has an improved spinning disk confocal with larger field-of-view, large field-of-view cameras, higher-power lasers, and optimized filter sets compared to the original system at CSHL. Furthermore, we also improved the data registration pipeline and imaging settings between collection of these two datasets. Thus, the differences in instrumentation and data processing alone could account for the improved data quality of the four littermate pairs. In addition, the pilot brain was collected at P56 and the four littermate pairs were collected at P28. Based on the current data, we could not distinguish whether the improved data quality was due to improvements in data collection and processing or due to differences in age.

Supplementary Note 7: The effect of enucleation on gene expression

We first examined whether the effect of enucleation was predominantly reflected in enrichment and/or depletion of H3 types that were already present in the control brains, or distinct H3 types in enucleated brains that were absent in the control brains. Consistent with the lack of batch effect seen in the UMAP plots (**Fig. 4D**), all four pairs of littermates had similar fractions of H1, H2, and cortical H3 types (**ED Fig 8D-F**). No H3 type was strongly enriched in either the control brains or the enucleated brains (**ED Fig 8F**). The lack of condition-specific H3 types can be visualized in UMAP plots, in which neurons color-coded by their conditions were smoothly intermixed together (**Fig. 4F**). This lack of new cell types is also reflected in the spatial distribution of H2 types, which were visually similar across the two conditions (**ED Fig. 8I**).

To test whether there are changes at a finer granularity than the H3 types, we identified the 100 nearest neighbors of each neuron based on gene expression, and asked what fraction of those neurons were from the control brains. For all neurons, 14 – 90% of neighboring neurons were from the control brains, which was consistent with the composition of H3 types. These results suggest that enucleation did not produce neighborhoods of neurons that were absent in the control animals.

We next tested if subpopulations of neurons within H3 types were preferentially enriched in neurons from the enucleated brains or the control brains by treating the kNN structure as a classifier: For each neuron, we predicted its condition of origin (control or enucleated) using its 100 closest neighbors (see **Methods**). If an H3 type or a subpopulation of an H3 type is seen only in the enucleated brain, then we would expect that neurons in that neighborhood to be surrounded by mostly neurons from the enucleated brains, thus resulting in a high AUROC score. In contrast, the classifier largely failed to predict whether a neuron was from a control or an enucleated brain (mean AUROC was 0.49). The classifier, however, was weakly predictive in seven areas (including visual areas and RSPagl) in at least 3 out of 4 litters (visual areas and RSPagl, **ED Fig. 8G**), with VISp showing the clearest difference (AUROC=0.61). Within H3 types, 12 combinations showed moderately positive performance using the neighbor-based classifier (AUROC > 0.6, **ED Fig. 8H**), all involving L2/3 or L4/5 IT types. This moderate performance suggests that either heterogenous subpopulations within these H3 types were non-uniformly depleted or enriched, or that enucleation created new cell states within the existing H3 types. Whether such heterogeneity reflects the creation of new cell types relies on distinguishing transcriptomic signatures of cell types versus cell states, which is hotly debated in the field and cannot be settled with our current dataset. Thus, we did not further analyze the within-H3 type shifts in gene expression.

Supplementary Note 8: Comparison to existing studies

Several studies have previously examined the effect of removing visual stimuli on gene expression and cell types in the mouse visual cortex. In these studies, effects varied according to the specifics of how visual inputs were removed. Removal of sensory input generally falls into two categories: blocking thalamic axons from reaching cortical targets, or removing sensory input from the thalamic projections via sensory deprivation after the axons have reached the cortex. This study (bilateral enucleation at P1) falls into the second category.

Two previous studies [40,42] perturbed visual thalamic inputs in ways that were most relevant to the perturbation we performed. Cheng, et al. [42] examined the effects of dark rearing during the visual critical period. This perturbation represented a similar but less severe sensory deprivation compared to bilateral enucleation performed in this study. Cheng, et al. [42] reported changes in gene expression in L2/3 IT neurons, corresponding to a change in cell type composition from three identified L2/3 IT cell types in visual cortex. To compare our results to their observations, we mapped H3 types in our dataset to cortical excitatory types in Cheng, et al. [42] (**ED Fig. 9C**) and found a strong correspondence across all types. For L2/3 IT neurons, three out of six H3 types in our data corresponded strongly to types L2/3 IT A, B, and C in Cheng, et al. [42]. What is more, the sub-laminar distribution of matched cell types (**ED Fig. 9B**) also matched across our study and Cheng, et al. [42]: Type L2/3 IT UL maps closely to type A in Cheng, et al. [42] and is in the superficial layer 2/3; L2/3 IT ML-2 corresponds to type B and is located in mid layer 2/3; L2/3 IT DL matches to both type B and C and is located in deep layer 2/3. Consistent with the findings of Cheng, et al. [42], we found that L2/3 IT ML-2 were reduced in VISp (**Fig. 5E**). In addition, we also observed broader changes in other IT neurons (L4/5 IT and L6 IT), which were not seen in Cheng, et al. [42]. We speculate that the broader changes seen in our data reflected the earlier developmental timepoint at which enucleation was performed. Because

IT neurons in deeper layers differentiate and presumably mature earlier, an earlier sensory deprivation would likely have a stronger effect on neurons in deeper layers than sensory deprivation during only the critical period. Thus, our data recapitulated similar changes in L2/3 IT as observed in Cheng, et al. [42], but also revealed broader changes in other cell types that likely resulted from sensory deprivation starting from an earlier age.

In a second study, Chou, et al. [40] used a conditional knockout mutant to prevent most axons from the lateral geniculate nucleus (LGN) to reach the visual cortex during development, which eliminated the difference between primary visual cortex and higher visual areas. This contrasts with our finding that the primary visual cortex remained distinct from secondary visual areas despite broad changes in H3 types. We speculate that these differences likely reflect the different types of perturbation performed in the two studies: Whereas Chou, et al. [40] blocked thalamic axons from reaching the cortex, our study only blocked sensory activity in those axons after they reached the cortex. These differences suggest that the physical connections established by thalamocortical axons are needed to define the primary visual cortex, and that the sensory activity conveyed through these axons plays a refinement role in cell type composition across both the primary visual cortex and neighboring higher visual areas.

	Olympus IX81				Nikon Ti-2E (no excitation filter)		
Channel	Laser	Excitation filter	Dichroic	Emission filter	Laser	Dichroic	Emission filter
G/YFP	520	zet443-518x	zt443-518rpc	FF01-565/24	514	Zt405/514/635rpc	FF01-565/24
T	555	zet402/468/555/640x	zt402/468/555/640rpc-ufs	ff01-585/11	561	FF421/491/567/659/776-Di01	FF01-441/511/593/684/817
A	640	zet402/468/555/640x	FF652-Di01	FF01-676/29	640	Zt405/514/635rpc	FF01-676/29
C	640	zet402/468/555/640x	FF652-Di01	FF01-725/40	640	Zt405/514/635rpc	FF01-775/140
GFP	470	zet402/468/555/640x	zt402/468/555/640rpc-ufs	FF01-525/30	488	FF421/491/572-Di01	69401m
DAPI	405	zet402/468/555/640x	zt402/468/555/640rpc-ufs	69401m	405	FF421/491/572-Di01	69401m
TxRed	555	zet402/468/555/640x	zt402/468/555/640rpc-ufs	69401m	561	FF421/491/572-Di01	69401m
Cy5	640	zet402/468/555/640x	FF652-Di01	FF01-676/29	640	Zt405/514/635rpc	ZET532/640m

Supplementary Table 2. Microscopy filters and lasers used for BARseq

Supplementary references

68 Fischer, S. & Gillis, J. How many markers are needed to robustly determine a cell's type? *iScience* **24**, 103292 (2021). **https://doi.org:10.1016/j.isci.2021.103292**

69 Puchades, M. A., Csucs, G., Ledergerber, D., Leergaard, T. B. & Bjaalie, J. G. Spatial registration of serial microscopic brain images to three-dimensional reference atlases with the QuickNII tool. *PLoS One* **14**, e0216796 (2019). **https://doi.org:10.1371/journal.pone.0216796**

70 Wang, X. *et al*. Three-dimensional intact-tissue sequencing of single-cell transcriptional states. *Science* (2018). **https://doi.org:10.1126/science.aat5691**

71 Meng, X., Winkowski, D. E., Kao, J. P. Y. & Kanold, P. O. Sublaminar Subdivision of Mouse Auditory Cortex Layer 2/3 Based on Functional Translaminar Connections. *J Neurosci* **37**, 10200-10214 (2017). **https://doi.org:10.1523/JNEUROSCI.1361-17.2017**
