# Supplementary material for: Whole-cortex in situ sequencing reveals input-dependent area identity
Source: Nature. 2024 Apr 24;647(8088):203–12. doi: 10.1038/s41586-024-07221-6 (PMC12589132; doi:10.1038/s41586-024-07221-6)
Supplement: Supplementary file 2 — Reporting Summary [file 41586_2024_7221_MOESM2_ESM.pdf]

nature portfolio

Corresponding author(s): Xiaoyin Chen, Jesse Gillis, Anthony Zador

Last updated by author(s): Jan 22, 2024

# Reporting Summary

Nature Portfolio wishes to improve the reproducibility of the work that we publish. This form provides structure for consistency and transparency in reporting. For further information on Nature Portfolio policies, see our Editorial Policies and the Editorial Policy Checklist.

## Statistics

For all statistical analyses, confirm that the following items are present in the figure legend, table legend, main text, or Methods section.

| n/a | Confirmed | |
|---|---|---|
| ☐ | ☒ | The exact sample size (*n*) for each experimental group/condition, given as a discrete number and unit of measurement |
| ☐ | ☒ | A statement on whether measurements were taken from distinct samples or whether the same sample was measured repeatedly |
| ☐ | ☒ | The statistical test(s) used AND whether they are one- or two-sided<br>*Only common tests should be described solely by name; describe more complex techniques in the Methods section.* |
| ☐ | ☒ | A description of all covariates tested |
| ☐ | ☒ | A description of any assumptions or corrections, such as tests of normality and adjustment for multiple comparisons |
| ☐ | ☒ | A full description of the statistical parameters including central tendency (e.g. means) or other basic estimates (e.g. regression coefficient) AND variation (e.g. standard deviation) or associated estimates of uncertainty (e.g. confidence intervals) |
| ☐ | ☒ | For null hypothesis testing, the test statistic (e.g. *F*, *t*, *r*) with confidence intervals, effect sizes, degrees of freedom and *P* value noted<br>*Give P values as exact values whenever suitable.* |
| ☒ | ☐ | For Bayesian analysis, information on the choice of priors and Markov chain Monte Carlo settings |
| ☒ | ☐ | For hierarchical and complex designs, identification of the appropriate level for tests and full reporting of outcomes |
| ☐ | ☒ | Estimates of effect sizes (e.g. Cohen's *d*, Pearson's *r*), indicating how they were calculated |

*Our web collection on statistics for biologists contains articles on many of the points above.*

## Software and code

Policy information about availability of computer code

| | |
|---|---|
| Data collection | Data collection used micro-manager v1.4 and NIS-Elements AR (5.30.04) to drive microscope |
| Data analysis | Custom R (v4.3.0), python(3.8 for cellpose and n2v, 3.9 for bardensr), and MATLAB (2023a) codes were used to process and analyze data. These codes relied on open-source packages, including Bioconductor(v3.18), Cellpose(2.2), Bardensr, n2v(0.3.1), QuickNii(2.2), Visualign(0.9), and FIJI (1.53t). Custom codes are provided on Mendeley Data (https://data.mendeley.com/datasets/8bhhk7c5n9/1 and https://data.mendeley.com/datasets/5xfzcb4kn8/1) and on Github (https://github.com/gillislab/barseq_analysis). |

For manuscripts utilizing custom algorithms or software that are central to the research but not yet described in published literature, software must be made available to editors and reviewers. We strongly encourage code deposition in a community repository (e.g. GitHub). See the Nature Portfolio guidelines for submitting code & software for further information.

## Data

Policy information about availability of data

All manuscripts must include a data availability statement. This statement should provide the following information, where applicable:

- Accession codes, unique identifiers, or web links for publicly available datasets
- A description of any restrictions on data availability
- For clinical datasets or third party data, please ensure that the statement adheres to our policy

Raw sequencing images are available from the Brain Image Library (https://api.brainimagelibrary.org/web/view?bildid=ace-dim-pad, https://

api.brainimagelibrary.org/web/view?bildid=ace-dim-own, https://api.brainimagelibrary.org/web/view?bildid=ace-dim-owl, https://api.brainimagelibrary.org/web/view?bildid=ace-dim-out, https://api.brainimagelibrary.org/web/view?bildid=ace-dim-orb, https://api.brainimagelibrary.org/web/view?bildid=ace-dim-old, https://api.brainimagelibrary.org/web/view?bildid=ace-dim-off, https://api.brainimagelibrary.org/web/view?bildid=ace-dim-odd, https://api.brainimagelibrary.org/web/view?bildid=ace-cry-zip). Cell-level and rolony-level data are provided at Mendeley data (https://data.mendeley.com/datasets/8bhhk7c5n9/1 and https://data.mendeley.com/datasets/5xfzcb4kn8/1). Gene panel selection and cell type assessment were based on public data available at https://data.nemoarchive.org/biccn/lab/zeng/transcriptome/ and https://github.com/shekharlab/mouseVC. Allen CCF v3 with the 2017 annotation was downloaded from http://ihelp.corp.alleninstitute.org/display/mouseconnectivity/API#API-InformaticsDataProcessing.

## Research involving human participants, their data, or biological material

Policy information about studies with human participants or human data. See also policy information about sex, gender (identity/presentation), and sexual orientation and race, ethnicity and racism.

| | |
|---|---|
| Reporting on sex and gender | NA |
| Reporting on race, ethnicity, or other socially relevant groupings | NA |
| Population characteristics | NA |
| Recruitment | NA |
| Ethics oversight | NA |

Note that full information on the approval of the study protocol must also be provided in the manuscript.

# Field-specific reporting

Please select the one below that is the best fit for your research. If you are not sure, read the appropriate sections before making your selection.

☒ Life sciences ☐ Behavioural & social sciences ☐ Ecological, evolutionary & environmental sciences

For a reference copy of the document with all sections, see nature.com/documents/nr-reporting-summary-flat.pdf

# Life sciences study design

All studies must disclose on these points even when the disclosure is negative.

| | |
|---|---|
| Sample size | Sample sizes were chosen to include duplicates per sex per condition (8 animals), plus an additional brain for pilot study. We saw that the changes associated with enucleation were much stronger than inter-individual variations across replicates/sexes, indicating that our sample size was sufficient. |
| Data exclusions | Cells with low read counts and gene counts were excluded, because they would not be robustly clustered. For part of the analyses, coronal sections from the most anterior and posterior end of the cortex were excluded. These sections were excluded because coronal cuts were not perpendicular to the cortex due to the curvature of the cortex, and so we cannot reliably estimate cell type composition in cubelets drawn on slices. This criteria was pre-established based on the anatomy of the cortex. |
| Replication | The experiments were replicated on four littermate pairs, and the observed effects were consistent across all four replicates. The cell typing results were consistent across all eight littermates and also between the littermates and the pilot brain.All attempted replicates were included in the paper. |
| Randomization | Littermates were randomly assigned to either enucleated or sham condition. |
| Blinding | Because the enucleated animals were easily distinguishable from control animals during both the experiment and data analysis, we did not attempt to blind the experiment during both data collection and analysis. The systematic and comprehensive analysis we performed were not prone to observer biases and thus did not require blinding. This approach conforms with standard practice for -omics studies. |

# Reporting for specific materials, systems and methods

We require information from authors about some types of materials, experimental systems and methods used in many studies. Here, indicate whether each material, system or method listed is relevant to your study. If you are not sure if a list item applies to your research, read the appropriate section before selecting a response.

## Materials & experimental systems

| n/a | Involved in the study |
|---|---|
| ☒ | ☐ Antibodies |
| ☒ | ☐ Eukaryotic cell lines |
| ☒ | ☐ Palaeontology and archaeology |
| ☐ | ☒ Animals and other organisms |
| ☒ | ☐ Clinical data |
| ☒ | ☐ Dual use research of concern |
| ☒ | ☐ Plants |

## Methods

| n/a | Involved in the study |
|---|---|
| ☒ | ☐ ChIP-seq |
| ☒ | ☐ Flow cytometry |
| ☒ | ☐ MRI-based neuroimaging |

# Animals and other research organisms

Policy information about studies involving animals; ARRIVE guidelines recommended for reporting animal research, and Sex and Gender in Research

| | |
|---|---|
| Laboratory animals | C57BL/6J male and female 4-8 weeks old mice were used |
| Wild animals | The study did not involve wild animals |
| Reporting on sex | The pilot study involved a male mouse. For experiments in which one littermate each out of four pairs was enucleated, we matched the sex of each littermate pair and analyzed data from four female and four male mice, two enucleated and two control mice of each sex. |
| Field-collected samples | The study did not involve field-collected samples |
| Ethics oversight | All animal procedures were carried out in accordance with the Institutional Animal Care and Use Committee at Cold Spring Harbor Laboratory and John Hopkin's University. |

Note that full information on the approval of the study protocol must also be provided in the manuscript.

# Plants

| | |
|---|---|
| Seed stocks | NA |
| Novel plant genotypes | NA |
| Authentication | NA |
